# Association of inflammatory markers with survival in patients with advanced gastric cancer treated with immune checkpoint inhibitors combined with chemotherapy as first line treatment

**DOI:** 10.3389/fonc.2022.1029960

**Published:** 2022-10-28

**Authors:** Mingyu Wan, Yongfeng Ding, Chenyu Mao, Xiaolu Ma, Ning Li, Cheng Xiao, Jiong Qian, Haiping Jiang, Yulong Zheng, Luntao Wu, Lisong Teng, Nong Xu

**Affiliations:** ^1^ Department of Medical Oncology, The First Affiliated Hospital of Zhejiang University, Hangzhou, China; ^2^ Department of Surgical Oncology, The First Affiliated Hospital of Zhejiang University, Hangzhou, China

**Keywords:** neutrophil-to-lymphocyte ratio, immune checkpoint inhibitor, inflammatory markers, gastric cancer, prognosis

## Abstract

**Background:**

The emergence of immune checkpoint inhibitors has changed the landscape of first-line treatment of patients with advanced gastric cancer. Currently, the prognostic significance of inflammatory markers in first-line immunotherapy combined with chemotherapy for gastric cancer is currently unclear. This study aimed to identify inflammatory markers with potential to predict treatment outcome in advanced gastric cancer patients receiving immunotherapy combined with chemotherapy.

**Methods:**

This retrospective study enrolled untreated advanced or metastatic gastric or gastro-esophageal junction cancer patients from 5 clinical trials (the clinical trial cohort) and the real world (the real-word cohort). Inflammatory markers included in the analysis included neutrophil-to-lymphocyte ratio (NLR), monocyte-to-lymphocyte ratio (MLR), platelet-to-lymphocyte ratio (PLR), systemic inflammation index (SII), and derived neutrophil-to-lymphocyte ratio (dNLR). Receiver operating characteristic (ROC) curves were constructed to identify optimal cut-off values. The prognostic potential of the markers was determined using Kaplan–Meier analysis, univariate and multivariate Cox-regression analyses in the clinical trial cohort and the findings were validated in the real-world cohort.

**Results:**

In the clinical trial cohort (n=45), MLR, PLR and SII were associated with PFS but not OS (All P<0.05), while dNLR was not correlated with PFS or OS. Only NLR was associated with PFS and OS and identified as an independent prognostic predictor in the univariate and multivariate analyses. The prognostic value of NLR was validated in the real-world cohort (n=55).

**Conclusions:**

NLR was a strong predictor of PFS and OS in patients with advanced gastric cancer receiving immune checkpoint inhibitors combined with chemotherapy. Further prospective studies are required to validate our results.

## Introduction

Gastric cancer (GC) is the fourth leading cause of cancer-related deaths worldwide, with over one million new cases and 769,000 deaths reported in 2020 (1). Although, doublet fluoropyrimidine-based and platinum-based chemotherapy regimens are the preferred first-line treatments, immune checkpoint inhibitors in combination with chemotherapy are becoming the standard of care for patients with advanced gastric cancer (2–4). The 2-year follow-up data of the CheckMate 649 trial published at the 2021 ESMO Annual Meeting revealed that nivolumab combined with chemotherapy resulted in a longer OS (13.8 vs.11.6 months, HR: 0.80; P≥ 0.0002) compared with chemotherapy alone (5). As a result, the U.S. Food and Drug Administration (FDA) approved nivolumab in combination with chemotherapy in April 2021 as the first-line treatment for metastatic gastric cancer and esophageal adenocarcinoma. A recent study showed that the combination of sintilimab plus chemotherapy improved the OS and PFS regardless of PD-L1 expression status (15.2 vs.12.3 months; HR: 0.766; P ≥ 0.009) (6). One of the hallmarks of immunotherapy is its long-term efficacy, making it desirable to identify non-responders.

Several biomarkers such as PD-L1 expression (7), tumor mutation burden(TMB) (8) and microsatellite instability (MSI) have been considered to be potential predictors of survival outcomes (9). Unfortunately, the prognostic value of baseline biomarkers remains unclear. Currently, evidence from previous studies has linked inflammation to the proliferation, survival, and migration of tumor cells (10, 11). The causal link between inflammation and cancer is now well demonstrated (12, 13). The significance of inflammatory markers in predicting the outcome of patients who receive immune checkpoint inhibitors has been investigated (14). Inflammatory markers, such as neutrophil-to-lymphocyte ratio (NLR), monocyte-to-lymphocyte ratio (MLR), platelet-to-lymphocyte ratio (PLR), systemic inflammation index (SII), and derived neutrophil-to-lymphocyte ratio (dNLR), are potential predictors of immunotherapy efficacy in different cancers (15–17), suggesting that their analogies may have broad clinical applications. However, few studies have explored the function of inflammatory markers in patients with advanced gastric cancers receiving immunotherapy combined with chemotherapy, particularly those receiving the combination as first line treatment.

In this study, we explored the ability of inflammatory markers to predict the efficacy of immune checkpoint inhibitors combined with chemotherapy as first-line therapy for patients with advanced gastric cancer. The association between the inflammatory markers and survival was examined in a clinical trial cohort. The results were validated in an independent, real-world cohort, with the aim of identifying potential prognostic markers.

## Methods

### Study design and patients

This retrospective study enrolled patients diagnosed with advanced or metastatic gastric or gastro-esophageal junction cancer at the Medical Oncology Department of the First Affiliated Hospital of Zhejiang University. The inclusion criteria were as follows: histologically proven gastric adenocarcinoma; stage III or IV; received no previous systemic treatment or received prior neoadjuvant/adjuvant therapy if completed ≥6 months; at least six cycles of combined therapy and one treatment response evaluation; Eastern Cooperative Oncology Group (ECOG) PS ≤ 2. The exclusion criteria were as follows: pre-treatment blood count values were not obtained two weeks before the treatment initiation; did not receive immunotherapy as the initial therapy; double primary cancers; recent operation; infectious diseases; missing medical records of the first cycle; missing data on blood count. All patients received immune checkpoint inhibitors combined with chemotherapy as first-line treatment. All included patients with recurrence underwent R0 resection previously. The chemotherapy regimens were as follows: oxaliplatin plus capecitabine (XELOX), S-1 plus paclitaxel (SPA), oxaliplatin plus fluorouracil and leucovorin (FOLFOX) and S-1 plus oxaliplatin (SOX). The choice of combined regimen and dosage was based on the actual condition and preference of patients.

The clinical trial cohort comprised 45 patients from 5 clinical trials conducted in the Medical Oncology Department of the First Affiliated Hospital of Zhejiang University. The treatment was initiated from December 2017 to December 2020. Part of these clinical trials had previously been reported (18–21). The real-world cohort included 55 patients from the Medical Oncology Department. The treatment was initiated from March 2018 to June 2021. The study was approved by the Ethics Committee of Zhejiang University (reference number: 2022–375).

### Data collection

Clinicopathological characteristics and follow-up status were collected. All laboratory values were measured two weeks before treatment initiation. Data were collected from the electronic medical records system. Fasting venous blood was collected in the early morning and analyzed. Staging evaluation was based on the eighth edition of TMN staging published by the International Cancer Control Alliance. Computed tomography (CT) and/or magnetic resonance imaging (MRI) were performed at baseline and every 2 or 3 cycles. Clinical response was classified as complete remission (CR), partial remission (PR), stable disease (SD), and progressive disease (PD), according to the Response Evaluation Criteria in Solid Tumors (RECIST [version 1.0]). Human epidermal growth factor receptor-2 (HER-2) was defined as positive if the IHC score was 3+ or if fluorescence *in situ* hybridization (FISH) was positive. NLR, MLR, PLR, SII and dNLR were calculated using the formula: neutrophils/lymphocytes, monocytes/lymphocytes, platelets/lymphocytes, neutrophils*platelets/lymphocytes, and neutrophils/(leukocytes-neutrophils), respectively.

### Statistical analysis

Baseline characteristics of patients were summarized using descriptive frequencies and percentages, and categorical variables were analyzed using the Chi-square test or Fisher’s exact test. P value < 0.05 indicated statistically significant differences. Receiver operating characteristics (ROC) curve analysis was performed to determine the optimal cutoff value for each parameter. Survival curves were estimated using Kaplan-Meier analysis and differences were compared using log-rank test. The median estimated follow-up was calculated using the reverse Kaplan-Meier method, while univariate and multivariate Cox regression analyses were conducted to identify independent predictors of PFS and OS. Factors significantly associated with the PFS and OS in the univariable analysis were included in the multivariate analysis based on the stepwise forward procedure with enter and remove limits of 0.05 and 0.10, respectively. OS was defined as the time between the start of combined therapy and death or the last follow-up, while PFS was defined as the time between the start of combined therapy and disease progression or death. Statistical analyses were conducted on GraphPad Prism 9.0 (GraphPad Software, La Jolla, CA, USA), R language (R Core Team) and SPSS 26.0 (IBM Corp., Armonk, NY, USA).

## Results

### Characteristics of patients (clinical trial cohort)

A total of 45 patients were enrolled in the clinical trial cohort and their baseline characteristics are shown in **Table 1**. The age of the patients ranged from 37 to 74 years, with a median age of 64 years. Among them, 35 (78%) patients were male, 35 (78%) patients were ECOG PS 0, and 9 (20%) patients had cancer of GEJ. Notably, 8 (18%) of the patients had stage III whereas 37 (82%) had stage IV. In addition, 39 (100%, 39 of 39) patients were HER2-negative, while the HER2 status of the remaining patients was undetermined. Moreover, 44 (98%) patients were treated with capecitabine and oxaliplatin (XELOX) and only 1(2%) patient was treated with oxaliplatin plus fluorouracil and leucovorin (FOLFOX). Overall, 44 (98%) patients received an anti-PD-1 antibody treatment and only 1(2%) patient received an anti-PD-L1 antibody treatment (**Supplementary Table S1**).

**Table 1:** Clinical characteristics of the clinical trial and real-world cohort.

Characteristics	Clinical trial cohort (n = 45) n(%)	Real-world cohort (n = 55) n(%)	P-value
Age			0.364
<60	34 (76)	37 (67)	
≥60	11 (24)	18 (33)	
Sex			0.867
Male	35 (78)	42 (76)	
Female	10 (22)	13 (24)	
ECOG PS			0.001*
0	35 (78)	25 (45)	
1	10 (22)	25 (45)	
2	0 (0)	5 (9)	
Location			0.118
GEJ	9 (20)	5 (9)	
Non-GEJ	36 (80)	50 (91)	
Stage			0.108
III	8 (18)	4 (7)	
IV	37 (82)	51 (93)	
Lauren classification			0.550
Intestinal type	20 (61)	10 (59)	
Diffused type	6 (18)	5 (29)	
Mixed type	7 (21)	2 (12)	
Unknown	12	38	
HER2 expression			0.494*
Positive	0 (0)	2 (5)	
Negative	39 (100)	40 (95)	
Unknown	6	13	
Metastasis site			0.036*
Liver	22 (49)	24 (44)	
Peritoneal	6 (13)	13 (24)	
Bone	6 (13)	1 (2)	
Pancreas	0 (0)	5 (9)	
Abdominal wall	0 (0)	5 (9)	
Colorectal	1 (2)	2 (4)	
Ovary	1 (2)	2 (4)	
Bladder	2 (4)	1 (2)	
Others	5 (11)	6 (11)	
MMR status			0.485*
pMMR	31 (97)	34 (100)	
dMMR	1 (3)	0 (0)	
Unknown	13	21	
PD-L1 expression			1*
Positive	8 (42)	0 (0)	
Negative	11 (58)	1 (100)	
Unknown	26	54	
Differentiation			0.015
Poor	15 (39)	31 (66)	
Moderate-well	23 (61)	16 (34)	
Unknown	7	8	
History of gastric cancer operation			0.110
Yes	7 (16)	16 (29)	
No	38 (84)	39 (71)	
History of smoke			0.099
Yes	10 (23)	21 (38)	
No	34 (77)	34 (62)	
Unknown	1	0	
History of alcohol			0.475
Yes	10 (23)	16 (29)	
No	34 (77)	39 (71)	
Unknown	1	0	

ECOG PS, Eastern Cooperative Oncology Group Performance Status; MMR, proficient mismatch; pMMR, proficient mismatch repair; dMMR, deficient mismatch repair.

*Fisher’s exact probability method.

### Efficiency (clinical trial cohort)

At the cutoff date of August 1, 2021, the median estimated follow-up time was 27.3 (95% CI 25.9-28.7) months. The median PFS and OS were 10.0 (95% CI 26.2-13.7) months and 17.7 (95% CI 29.4-26.0) months (**Supplementary Figures S1A, B**), respectively. There were 8 (17.7%) cases of CR, 25 (55.5%) cases of PR, 11 (24.4%) cases of SD, and 1 (2.2%) case of PD among the 45 patients. The overall response rate (ORR) was 73.3% and the disease control rate (DCR) was 97.7%. The 1-year survival rate was 62.2% (**Supplementary Table S2**).

### Prognostic significance of inflammatory markers (clinical trial cohort)

**Figure 1:**
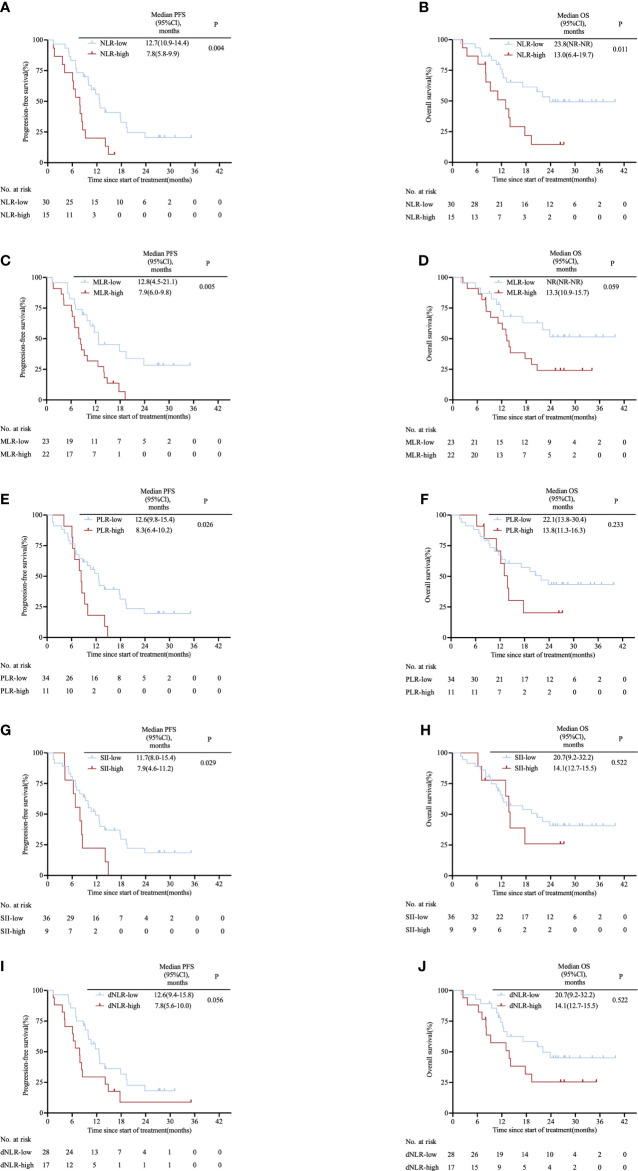
Kaplan–Meier survival curves in the clinical trial cohort stratified by inflammatory markers for **(A)** PFS for NLR cut-off, **(B)** OS for NLR cut-off, **(C)** PFS for MLR cut-off, **(D)** OS for MLR cut-off, **(E)** PFS for PLR cut-off, **(F)** OS for PLR cut-off, **(G)** PFS for SII cut-off, **(H)** OS for SII cut-off, **(I)** PFS for dNLR cut-off and **(J)** OS for dNLR cut-off. The p values were calculated using the log-rank test (two-sided). *CI*, confidence interval*; dNLR*, derived neutrophil-to-lymphocyte ratio*; MLR*, monocyte-to-lymphocyte ratio*; NLR*, neutrophil-to-lymphocyte ratio*; OS*, overall survival*; PLR*, platelet-to-lymphocyte ratio*; PFS*, progression-free survival*; SII*, systemic immune-inflammation index.

The area under the ROC curve (AUC) was 0.683 for NLR, 0.660 for MLR, 0.558 for PLR, 0.662 for SII and 0.697 for dNLR (**Supplementary Figure S2**). Based on the cut-off value of inflammatory markers obtained from the ROC curves, the patients were divided into NLR-high (≥3.85) and NLR-low (<3.85); MLR-high (≥0.35) and MLR-low (<0.35); PLR-high (≥214.08) and PLR-low (<214.08); SII-high (≥1154.67) and SII-low (<1154.67); dNLR-high (≥2.45) and dNLR-low (<2.45) groups. **Figure 1** shows the survival curves grouped according to levels of inflammatory markers. Compared with patients with high levels of inflammatory markers, patients with low levels of inflammatory markers had a longer median PFS when divided by NLR (12.7 vs. 7.8 months; HR: 0.40, 95% CI 0.18–0.90; P= 0.004; **Figure 1A**), MLR (12.8 vs. 7.9 months; HR: 0.42, 95% CI 0.21–0.82; P= 0.005; **Figure 1C**), PLR (12.6 vs. 8.3 months; HR: 0.47, 95% CI 0.19–1.11; P= 0.026; **Figure 1E**), SII (11.7 vs. 7.9 months; HR: 0.45, 95% CI 0.17–1.18; P= 0.029; **Figure 1G**). Median PFS time was only comparable between low-dNLR group and high-dNLR group (12.6 vs. 7.8 months; HR: 0.53, 95% CI 0.26–1.11; P= 0.056; **Figure 1I**). There was no significant difference in OS among groups separated by MLR (NR vs. 13.3 months; HR: 0.48, 95% CI 0.22–1.04; P= 0.059; **Figure 1D**), PLR (22.1 vs. 13.8 months; HR: 0.61, 95% CI 0.24–1.56; P= 0.233; **Figure 1F**), SII (20.7 vs.14.1 months; HR: 0.74, 95% CI 0.28–2.01; P= 0.522; **Figure 1H**), and dNLR (22.1 vs. 13.8 months; HR: 0.54, 95% CI 0.23–1.23; P= 0.107; **Figure 1J**). Remarkably, lower NLR predicted a better OS (23.8 vs. 13.0 months; HR: 0.39, 95% CI 0.16–0.94; P= 0.011; **Figure 1B**). We also observed that NLR was still significantly associated with PFS (17.9 vs. 10.6 vs. 7.8 months; P= 0.005; **Supplementary Figure S3A**) and OS (NR vs. 20.7 vs. 13.0 months; P= 0.024; **Supplementary Figure S3B**) when patients were equally divided into three groups.

### NLR predicts outcomes in advanced gastric cancer patients undergoing immune checkpoint inhibitors combined with chemotherapy (clinical trial cohort)

Results of the univariate and multivariate proportional hazard analyses performed between baseline clinicopathological characteristics and survival in the clinical trial cohort are summarized in **Table 2**. Univariate predictors of PFS were age (HR: 0.48, 95% CI 0.24–0.95; P=0.036), ECOG PS (HR: 2.55, 95% CI 1.20–5.44; P=0.015), NLR (HR: 2.77, 95% CI 1.35–5.69; P=0.005), MLR (HR: 2.64, 95% CI 1.30–5.37; P=0.007), PLR (HR: 2.30, 95% CI 1.08–4.86; P=0.03) and SII (HR: 2.34, 95% CI 1.07–5.14; P=0.034), while ECOG PS (HR: 2.39, 95% CI 1.03–5.51; P=0.042) and NLR (HR: 2.59, 95% CI 1.20–5.61; P=0.016) were identified as independent predictors of PFS in the multivariate analysis. Similarly, univariate proportional hazard analyses revealed that NLR (HR: 2.66, 95% CI 1.21–5.82; P=0.015) was associated with OS and we found that NLR (HR: 3.35, 95% CI 1.42–7.91; P=0.006) along with ECOG PS (HR: 2.52, 95% CI 1.00–6.32; P=0.049) were independent predictors of OS (**Table 2**). Based on the prognostic parameters in the clinical trial cohort, we created a nomogram for predicting 1-year, 2-year, and median overall survival, the nomogram yielded an averaged concordance index (C-index) of 0.609 (95% CI, 0.491–0.727; **Supplementary Figure S4**).

**Table 2:** Univariate and multivariate analyses of the association between baseline characteristics and survival in the clinical trial cohort.

Variable	PFS		OS	
	Univariate analysis	Multivariate analysis	Univariate analysis	Multivariate analysis
	HR (95% CI), P	HR (95% CI), P	HR (95% CI), P	HR (95% CI), P
Age (≥60 vs <60)	0.48 (0.24-0.95), P=0.036	P=0.400	0.67(0.31-1.49), P=0.329	–
Gender (female vs male)	1.66 (0.78-3.56), P=0.191	–	1.58(0.63-3.98), P=0.328	–
ECOG PS (1 vs 0)	2.55 (1.20-5.44), P=0.015	2.39(1.03-5.51), P=0.042	1.91(0.83-4.41), P=0.128	2.52(1.00-6.32), P=0.049
Liver metastasis (yes vs no)	0.87 (0.45-1.69), P=0.688	–	0.75(0.35-1.64), P=0.478	–
Peritoneum metastasis (yes vs no)	1.14 (0.44-2.98), P=0.784	–	1.33(0.46-3.87), P=0.600	–
Differentiation (poor vs moderation-well)	1.48 (0.72-3.05), P=0.292	P=0.620	1.63(0.72-3.69), P=0.241	P=0.391
History of operation (yes vs no)	1.72 (0.69-4.27), P=0.241	–	2.09(0.78-5.59), P=0.144	–
History of smoke (yes vs no)	1.48 (0.70-3.18), P=0.312	–	1.32(0.52-3.32), P=0.556	–
History of alcohol (yes vs no)	1.17 (0.53-2.58), P=0.703	–	1.32(0.52-3.32), P=0.556	–
NLR (≥3.85 vs <3.85)	2.77 (1.35-5.69), P=0.005	2.59(1.20-5.61), P=0.016	2.66(1.21-5.82), P=0.015	3.35(1.42-7.91), P=0.006
MLR (≥0.35 vs <0.35)	2.64 (1.30-5.37), P=0.007	P=0.760	2.11(0.96-4.68), P=0.065	–
PLR (≥214.08 vs <214.08)	2.30 (1.08-4.86), P=0.030	P=0.742	1.66(0.72-3.85), P=0.238	–
SII (≥1154.67 vs <1154.67)	2.34 (1.07-5.14), P=0.034	P=0.607	1.35(0.54-3.37), P=0.524	–
dNLR (≥2.45 vs <2.45)	1.91 (0.97-3.75), P=0.060	–	1.87(0.86-4.07), P=0.112	–

CI, confidence interval; dNLR, derived neutrophil-to-lymphocyte ratio; ECOG PS, Eastern Cooperative Oncology Group Performance Status; HR, hazard ratio; MLR, monocyte-to-lymphocyte ratio; NLR, neutrophil-to-lymphocyte ratio; OS, overall survival; PLR, platelet-to-lymphocyte ratio; PFS, progression-free survival; SII, systemic immune-inflammation index.

In addition, we examined the association between NLR and adverse events. Adverse events related to first-line treatment in the clinical trial cohort are listed in **Supplementary Table S3**. All 45(100%) patients experienced at least one adverse event and 31(68.9%) patients experienced grade 3 or higher treatment-related adverse effects. However, NLR-high was not associated with an increased incidence of high-grade adverse events (P = 0.519, chi-square test).

### Characteristics of patients (real-world cohort)

We then validated the results obtained from the clinical cohort in an independent, real-world cohort of 55 patients. Of the 126 patients who had initially been recruited, 72 patients were excluded due to short treatment duration (n=47), recent operation(n=3), double primary cancer (n=4), immunotherapy not applied in the first cycle (n=2), and pre-treatment blood count not measured in 2 weeks(n=15) (**Supplementary Figure S5**). The baseline characteristics of patients are presented in **Table 1**. The age of the patients ranged from 32 to 81 years, with a median age of 65 years. 42 (76%) patients were male, 25 (45%) patients had an ECOG PS of 0, 25 (45%) patients had an ECOG PS of 1 and 5 (9%) patients had an ECOG PS of 2. The proportions of patients with stage III and stage IV disease were 7% and 93%, respectively. Moreover, 5 (9%) of the patients had GEJ cancer whereas 40 (95%, 40 of 42) patients were HER-2-negative. All patients received anti-PD-1 antibodies. The immunotherapy and chemotherapy regimens administered are presented (**Supplementary Table S1**). During the follow-up period, 30 patients (79%, 30 of 38) received subsequent treatment after they had disease progression while receiving first-line treatment. 18 (60%, 18 of 30) patients received targeted therapy, no patients received radiotherapy. The details of the treatment regimen are shown in **Supplementary Table S4**.

### Efficiency (real-world cohort)

At the data cutoff date of February 1, 2022, the median estimated follow-up time was 15.3 (95%CI 11.4–19.2) months. Median PFS was 8.6 (95%CI 6.1–11.1) months and median OS was 18.2 (95%CI 17.1–19.4) months (**Supplementary Figures S1C, D**). There were 3 (5.5%) cases of CR, 27 (49.1%) cases of PR, 17 (30.9%) cases of SD and 8 (14.5%) cases of PD in 55 patients. The ORR was 54.5%, and the DCR was 85.5%. The 1-year survival rate was 54.5% (**Supplementary Table S2**).

### Validation of the prognostic significance of NLR (real-world cohort)

**Figure 2:**
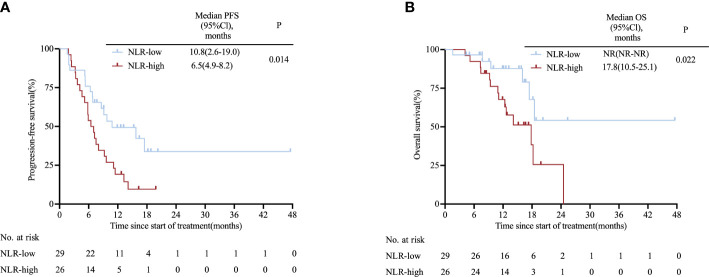
Kaplan-Meier survival curves in the real-world cohort stratified by NLR for **(A)** PFS for NLR cut-off and **(B)** OS for NLR cut-off. *CI*, confidence interval*; NLR*, neutrophil-to-lymphocyte ratio*; OS*, overall survival*; PFS*, progression-free survival.

Patients were divided into groups based on the same cut-off values used for the clinical trial cohort. A trend of superior PFS and OS benefit was consistently observed in NLR-low group compared with NLR-high group (10.8 vs. 6.5 months; HR: 0.46, 95% CI 0.24–0.88; P= 0.014; NR vs. 17.8 months; HR: 0.35, 95% CI 0.14–0.83; P= 0.022; **Figures 2A, B**). Additionally, we categorized patients into three groups equally and saw a similar trend in PFS (NR vs. 6.8 vs. 5.8 months; P= 0.016; **Supplementary Figure S6A**) and OS (18.5 vs. NR vs. 18.2 months; P= 0.08; **Supplementary Figure S6B**). Univariate proportional hazard analysis revealed that NLR, MLR and PLR were associated with PFS and OS. However, only high NLR (HR: 2.67, 95%CI 1.35–5.27; P= 0.005; HR: 3.69, 95% CI 1.40–9.11; P= 0.008) was found to be an independent predictor of poor outcomes in multivariate analysis (**Supplementary Table S5**). In addition, we validated our nomogram model with the real-world cohort, and the C-index was 0.669 (95% CI, 0.522–0.816), indicating good discrimination.

### Validation of the prognostic significance of NLR (pooled cohort)

**Figure 3:**
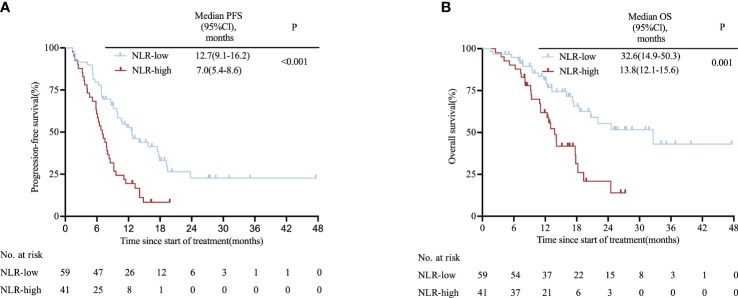
Kaplan-Meier survival curves in the pooled cohort stratified by NLR for **(A)** PFS for NLR cut-off and **(B)** OS for NLR cut-off. *CI*, confidence interval*; NLR*, neutrophil-to-lymphocyte ratio*; OS*, overall survival*; PFS*, progression-free survival.

The two cohorts were pooled to form one cohort for further analysis. Patients in the high-NLR group (n=41) had a shorter PFS than those in the low-NLR group (n=59) (7.0 vs. 12.7 months; HR: 2.30, 95% CI 1.40–3.78; P< 0.001; **Figure 3A**). Likewise, the high-NLR group had significantly shorter OS compared with those of the low-NLR group (13.8 vs. 32.6 months; HR: 2.50, 95% CI 1.36–4.60; P= 0.001; **Figure 3B**), with the 1-year OS rates for patients with low NLR and high NLR being 62.7% and 51.2%, respectively (**Supplementary Table S6**); These findings remained significant when we divided the pooled cohort equally into three groups according to NLR with respect to PFS (17.9 vs. 10.0 vs. 9.2 months; P= 0.002; **Supplementary Figure S7A**) and OS (32.6 vs. 20.7 vs. 13.0 months; P= 0.005; **Supplementary Figure S7B**). Results of Cox proportional hazard analysis confirm that NLR was an independent prognostic factor for PFS (HR: 2.75, 95% CI 1.61–4.71; P<0.001) and OS (HR: 3.25, 95% CI = 1.76–6.00; P<0.001) and validated to be an independent prognostic factor in multivariate analysis (**Supplementary Table S7**).

**Figure 4:**
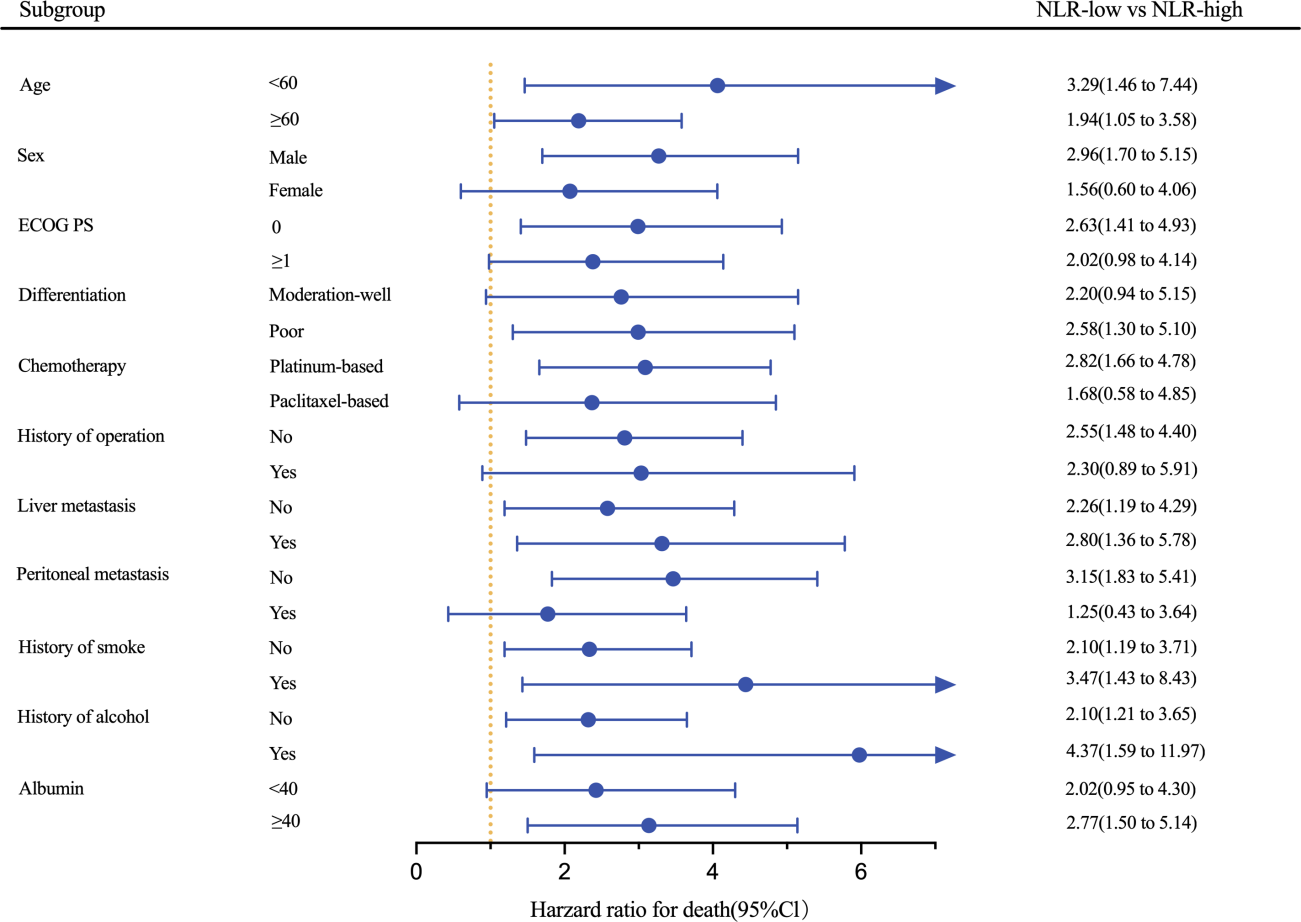
Forest plot of HRs for PFS based on the pooled cohort stratified by NLR cut-off. *ECOG PS*, Eastern Cooperative Oncology Group Performance Status*; NLR*, neutrophil-to-lymphocyte ratio.

Results of an exploratory analysis showed that the low-NLR group had longer PFS compared with the high-NLR group in most subgroups and significant improvement in PFS was achieved in subgroups irrespective of age, status of liver metastasis, history of smoke, and history of alcohol (all P<0.05; **Figure 4**). Among patients receiving platinum-based chemotherapy, those with low NLR had a better PFS (HR: 2.82, 95% CI 1.66–4.78; P<0.001). Furthermore, a trend of improvement was observed in PFS in other subgroups, although statistical significance was not achieved.

## Discussion

In the present study, we explored the role of inflammatory markers using a clinical trial cohort and validated the findings in a real-world cohort. Our principal finding was that NLR was the only independent inflammatory prognostic biomarker that showed a substantial effect on PFS and OS. Subgroup analysis in the pooled cohort based on age, liver metastasis, history of smoke and history of alcohol intake suggested that low NLR significantly prolonged PFS. To our knowledge, this is the first study to comprehensively assess the role of inflammatory markers in predicting the prognosis of previously untreated advanced gastric cancer patients receiving immune checkpoint inhibitors combined with chemotherapy.

More recent evidence has shown that inflammatory markers can predict short-term efficacy of immunotherapy in advanced gastric cancer patients. A study comprising 37 patients with advanced gastric cancer treated with first-line immunotherapy showed that PLR was an independent predictor of the OS but not the PFS (22). The baseline and early changes of MLR have been shown to influence survival outcomes in advanced gastric cancer on different lines (23). In our study, baseline MLR and PLR were associated with PFS, but not with OS. This discrepancy with previous reports may be attributed to confounding arising from the variety of lines and part of patients who received monotherapy. Remarkably, our study demonstrated that NLR was an independent prognostic factor for PFS and OS. Our results are consistent with those of previous studies, which demonstrate that NLR can predict the prognosis of several types of tumors (24). The relationship between elevated NLR and poor outcomes is supported by an umbrella review of 204 systematic reviews and meta-analyses from 86 studies (25). Several studies have recently demonstrated that NLR is an independent prognostic factor in patients with gastric or gastroesophageal junction cancer treated with nivolumab monotherapy (26–28). It has also been reported that the combination of NLR and TMB provides additional predictive value in patients treated with immunotherapy (29). The optimal cut-off of this research was defined as 3.85 for NLR by ROC analysis by nine-month progressive-free survival, which ranged from 2.5 to 4 reported in other studies (30–32). Given that the cut-off values of inflammatory markers are controversial, and our research provides a reference for future research. Interestingly, we found no significant association between NLR variations and ORR. It has been reported that NLR is not associated with ORR and DCR in patients receiving immunotherapy (33). Similarly, there was no statistical difference in ORR and DCR between low NLR and high NLR groups in 137 patients with metastatic gastric cancer treated with immunotherapy (34). A trend was found toward a higher CR rate in the NLR-low group (15.3% vs. 4.9%) and it required further investigation with larger sample sizes.

Of particular interest is the underlying basis of the relationship between NLR and outcomes. NLR characterizes the inflammatory response to cancer and reflects the disease burden. Neutrophils measured by NLR have been directly correlated with the intratumor neutrophil population (35). Tumor-activated neutrophils impair antitumor immunity and contribute to tumor progression *via* the GM-CSF-PD-L1 pathway (36) and engage in the metastatic process during cancer cell dissemination (37). Neutrophils are the primary source of metalloproteinase-9 (MMP-9), which promotes the release of vascular epithelial growth factor (VEGF) to create the tumor vasculature (38). Moreover, an elevated NLR is closely related to elevated circulating concentrations of inflammatory cytokines leading to a transition of the tumor microenvironment favorable for tumor invasion (39). Meanwhile, neutrophils have found to be associated with the myeloid-derived suppressor cells (MDSC) (40), which is associated with advanced status and metastases of various solid tumors (41). This may partly explain why high NLR always reflects a more advanced disease with potentially more aggressive tumors and is a potential prognostic factor for poor outcomes for patients with advanced gastric cancers (39). Cancer-triggered Immune response also relies on lymphocytes. Zhang et al. concluded that a high level of tumor-infiltrating lymphocytes (TIL) correlated with a low rate of metastasis and better survival in gastric cancer (42). The densities of CD3 (+), CD8 (+), and CD45RO (+) TILs were associated with lymph node metastasis and survival time (43). In a meta-analysis of 33 studies including 2559 patients, CD8+ TIL was an essential biomarker for predicting the efficacy of ICI in different cancers, regardless of monotherapy or combination with chemotherapy (44). Immune checkpoint inhibitors have been shown to improve outcomes of patients with advanced tumors, especially those infiltrated by CD8^+^T cells (45). Chemotherapeutics which induce immunogenicity (e.g., oxaliplatin, cyclophosphamide) can also provide additional or synergistic effects when used in combination with immune checkpoint inhibitors (46). High NLR indicates relatively depleted lymphocytes, leading to a weakened immune response to malignant cells (47, 48). These reasons may explain the unique prognostic value of NLR in patients treated with immune checkpoint inhibitors combined with chemotherapy. dNLR was calculated using the formulas: neutrophils/(leukocytes-neutrophils). Thus it does not reflect the level of lymphocytes well, which may be partially responsible for its ineffectiveness of prognostic value in advanced gastric cancer.

The exploration of prognostic markers based on clinical trials followed by validation in real-world settings provides reliable results with good consistency. Cuzick et al. developed a prognostic model using IHC markers in patients from the tamoxifen and anastrozole arms of the ATAC trial and validated their findings in an independent cohort in the real world (49). At present, most studies on inflammatory factors in gastric cancer treated with immunotherapy are based on single cohorts. Little focus has been paid to the identification of the function of MLR in patients using real-world cohorts (22). Based on results obtained from the clinical trial cohort, we concluded that NLR was an independent prognostic factor, a finding that was validated in the real-world cohort. Moreover, similar results were also found in the subgroup analysis in the pooled cohort. Therefore, the conclusions derived from this analysis are reliable.

However, our study had several limitations. First, this was a retrospective study involving patients from one study center. Second, the sample size of this study was small and data on the molecular characteristics of patients, such as major mismatch repair and PD-L1 expression, were insufficient. Therefore, it is difficult to draw a conclusion on the prognostic superiority NLR over the molecular indicators. Third, since the application time of combination therapy in the real world in China is relatively short, the median follow-up time in the real-world cohort is shorter than in the clinical trial cohort. Despite its limitations, this study demonstrates the potential of inflammatory markers to predict outcomes of patients with advanced gastric cancer treated with chemotherapy plus immunotherapy as first-line treatment. Future multicenter investigations are necessary to validate the results drawn from this research.

## Conclusion

In summary, we found that NLR can predict PFS and OS in patients with advanced gastric cancers who received first-line treatment of immunotherapy combined with chemotherapy. Therefore, NLR may become an inexpensive, usable, and reliable biomarker for predicting outcomes of patients with advanced gastric cancer in this setting. Further prospective studies should explore this possibility.

## Data availability statement

The raw data supporting the conclusions of this article will be made available by the authors, without undue reservation.

## Ethics statement

The studies involving human participants were reviewed and approved by Ethics Committee of Zhejiang University (reference number: 2022–375). Written informed consent for participation was not required for this study in accordance with the national legislation and the institutional requirements.

## Author contributions

MW and YD: data acquisition and analysis, writing. NL, XM, CX, JQ, HJ, YZ, and LW: data acquisition. YD and CM: review and editing. LT and NX: conceptualization, funding acquisition, review and editing. All authors read and approved the final manuscript.

## Funding

This work was supported by grants from National Health and Family Planning Commission Research Fund & Zhejiang Provincial Medical and Health Major Science and Technology Plan Project (No: KWJ-ZJ-1802).

## Conflict of interest

The authors declare that the research was conducted in the absence of any commercial or financial relationships that could be construed as a potential conflict of interest.

## Publisher’s note

All claims expressed in this article are solely those of the authors and do not necessarily represent those of their affiliated organizations, or those of the publisher, the editors and the reviewers. Any product that may be evaluated in this article, or claim that may be made by its manufacturer, is not guaranteed or endorsed by the publisher.

## References

[1] Sung H Ferlay J Siegel RL Laversanne M Soerjomataram I Jemal A. Global cancer statistics 2020: GLOBOCAN estimates of incidence and mortality worldwide for 36 cancers in 185 countries. CA Cancer J Clin (2021) 71(3):209–49. doi: 10.3322/caac.21660 33538338

[2] Shitara K Van Cutsem E Bang Y-J Fuchs C Wyrwicz L Lee K-W. Efficacy and safety of pembrolizumab or pembrolizumab plus chemotherapy vs chemotherapy alone for patients with first-line, advanced gastric cancer: The KEYNOTE-062 phase 3 randomized clinical trial. JAMA Oncol (2020) 6(10):1571–80. doi: 10.1001/jamaoncol.2020.3370 PMC7489405 32880601

[3] Moehler M Shitara K Garrido M Salman P Shen L Wyrwicz L. Nivolumab (nivo) plus chemotherapy (chemo) versus chemo as first-line (1L) treatment for advanced gastric cancer/gastroesophageal junction cancer (GC/GEJC)/esophageal adenocarcinoma (EAC): First results of the CheckMate 649 study. Ann Oncol (2020) 31:S1191–S. doi: 10.1016/j.annonc.2020.08.2296

[4] Boku N Ryu MH Oh DY Oh SC Chung HC Lee KW. Nivolumab plus chemotherapy versus chemotherapy alone in patients with previously untreated advanced or recurrent gastric/gastroesophageal junction (G/GEJ) cancer: ATTRACTION-4 (ONO-4538-37) study. Ann Oncol (2020) 31:S1192–S. doi: 10.1016/j.annonc.2020.08.2297

[5] Janjigian YY Ajani JA Moehler M Garrido M Gallardo C Shen L. Nivolumab (NIVO) plus chemotherapy (Chemo) or ipilimumab (IPI) vs chemo as first-line (1L) treatment for advanced gastric cancer/gastroesophageal junction cancer/esophageal adenocarcinoma (GC/GEJC/EAC): CheckMate 649 study. Ann Oncol (2021) 32:S1329–S30. doi: 10.1016/j.annonc.2021.08.2131

[6] Xu J Jiang H Pan Y Gu K Cang S Han L. Sintilimab plus chemotherapy (chemo) versus chemo as first-line treatment for advanced gastric or gastroesophageal junction (G/GEJ) adenocarcinoma (ORIENT-16): First results of a randomized, double-blind, phase III study. Ann Oncol (2021) 32:S1331–S. doi: 10.1016/j.annonc.2021.08.2133

[7] Gu LH Chen MM Guo DY Zhu HP Zhang WC Pan JH. PD-L1 and gastric cancer prognosis: A systematic review and meta-analysis. PloS One (2017) 12(8):1–14. doi: 10.1371/journal.pone.0182692 PMC5552131 28796808

[8] Greally M Chou JF Chatila WK Margolis M Capanu M Hechtman JF. Clinical and molecular predictors of response to immune checkpoint inhibitors in patients with advanced esophagogastric cancer. Clin Cancer Res (2019) 25(20):6160–9. doi: 10.1158/1078-0432.CCR-18-3603 PMC6905384 31337644

[9] Pietrantonio F Randon G Di Bartolomeo M Luciani A Chao J Smyth EC. Predictive role of microsatellite instability for PD-1 blockade in patients with advanced gastric cancer: a meta-analysis of randomized clinical trials. Esmo Open (2021) 6(1):1–5. doi: 10.1016/j.esmoop.2020.100036 PMC7815473 33460964

[10] Coussens LM Werb Z. Inflammation and cancer. Nature (2002) 420(6917):860–7. doi: 10.1038/nature01322 PMC2803035 12490959

[11] Mantovani A Allavena P Sica A Balkwill F. Cancer-related inflammation. Nature (2008) 454(7203):436–44. doi: 10.1038/nature07205 18650914

[12] Hanahan D Weinberg RA. Hallmarks of cancer: The next generation. Cell (2011) 144(5):646–74. doi: 10.1016/j.cell.2011.02.013 21376230

[13] Coussens LM Zitvogel L Palucka AK. Neutralizing tumor-promoting chronic inflammation: A magic bullet? Science (2013) 339(6117):286–91. doi: 10.1126/science.1232227 PMC3591506 23329041

[14] Mei Z Shi L Wang B Yang J Xiao Z Du P. Prognostic role of pretreatment blood neutrophil-to-lymphocyte ratio in advanced cancer survivors: A systematic review and meta-analysis of 66 cohort studies. Cancer Treat Rev (2017) 58:1–13. doi: 10.1016/j.ctrv.2017.05.005 28602879

[15] Diem S Schmid S Krapf M Flatz L Born D Jochum W. Neutrophil-to-Lymphocyte ratio (NLR) and platelet-to-Lymphocyte ratio (PLR) as prognostic markers in patients with non-small cell lung cancer (NSCLC) treated with nivolumab. Lung Cancer. (2017) 111:176–81. doi: 10.1016/j.lungcan.2017.07.024 28838390

[16] Jiang T Qiao M Zhao C Li XF Gao GH Su CX. Pretreatment neutrophil-to-lymphocyte ratio is associated with outcome of advanced-stage cancer patients treated with immunotherapy: a meta-analysis. Cancer Immunol Immunother (2018) 67(5):713–27. doi: 10.1007/s00262-018-2126-z PMC11028313 29423649

[17] Shang J Han X Zha HR Tao HT Li XY Yuan F. Systemic immune-inflammation index and changes of neutrophil-lymphocyte ratio as prognostic biomarkers for patients with pancreatic cancer treated with immune checkpoint blockade. Front Oncol (2021) 11. doi: 10.3389/fonc.2021.585271 PMC7943876 33718140

[18] Xu JM Xu N Bai YX Liu RR Mao CY Sui H. Anti-PD-1 antibody HX008 combined with oxaliplatin plus capecitabine for advanced gastric or esophagogastric junction cancer: a multicenter, single-arm, open-label, phase ib trial. Oncoimmunology (2021) 10(1):e1864908. doi: 10.1080/2162402X.2020.1864908 PMC7781732 33457083

[19] Jiang HP Zheng YL Qian J Mao CY Xu X Li N. Safety and efficacy of sintilimab combined with oxaliplatin/capecitabine as first-line treatment in patients with locally advanced or metastatic gastric/gastroesophageal junction adenocarcinoma in a phase ib clinical trial. BMC Cancer (2020) 20(1):760. doi: 10.1186/s12885-020-07251-z 32795349 PMC7427727

[20] Shen L Li J Xu N Xing B Zhang Q Zhao Y. A phase Ia/Ib trial of the anti-programmed death-ligand 1 (PD-L1) human monoclonal antibody (mAb), CS1001, in patients (pts) with advanced solid tumours or lymphomas. Ann Oncol (2019) 30:516–. doi: 10.1093/annonc/mdz253.093

[21] Yin XL Zhang Y Deng YH Xu N Xu JM Li L. Envafolimab plus chemotherapy in advanced gastric or gastroesophageal junction (G/GEJ) cancer. J Clin Oncol (2020) 38(15):e16585. doi: 10.1200/JCO.2020.38.15_suppl.e16585

[22] Qu ZT Wang QL Wang H Jiao Y Li M Wei W. The effect of inflammatory markers on the survival of advanced gastric cancer patients who underwent anti-programmed death 1 therapy. Front Oncol (2022) 12. doi: 10.3389/fonc.2022.783197 PMC8845037 35178344

[23] Chen Y Zhang C Peng Z Qi CS Gong JF Zhang XT. Association of lymphocyte-to-Monocyte ratio with survival in advanced gastric cancer patients treated with immune checkpoint inhibitor. Front Oncol (2021) 11. doi: 10.3389/fonc.2021.589022 PMC8203902 34141607

[24] Shaul ME Fridlender ZG. Tumour-associated neutrophils in patients with cancer. Nat Rev Clin Oncol (2019) 16(10):601–20. doi: 10.1038/s41571-019-0222-4 31160735

[25] Cupp MA Cariolou M Tzoulaki I Aune D Evangelou E Berlanga-Taylor AJ. Neutrophil to lymphocyte ratio and cancer prognosis: an umbrella review of systematic reviews and meta-analyses of observational studies. BMC Med (2020) 18(1):360. doi: 10.1186/s12916-020-01817-1 33213430 PMC7678319

[26] Ota Y Takahari D Suzuki T Osumi H Nakayama I Oki A. Changes in the neutrophil-to-lymphocyte ratio during nivolumab monotherapy are associated with gastric cancer survival. Cancer Chemotherapy Pharmacol (2020) 85(2):265–72. doi: 10.1007/s00280-019-04023-w 31907646

[27] Yamada T Hayashi T Inokuchi Y Hayashi K Watanabe H Komori K. Impact of the neutrophil-to-Lymphocyte ratio on the survival of patients with gastric cancer treated with nivolumab monotherapy. Targeted Oncol (2020) 15(3):317–25. doi: 10.1007/s11523-020-00716-y 32319020

[28] Fujita K Haruki N Kurehara H Ochi N Yamakawa Y Harata S. Neutrophil-lymphocyte ratio as a prognostic indicator in patients treated with nivolumab for gastric cancer. Gan to kagaku ryoho Cancer chemotherapy. (2020) 47(6):923–6. 32541169

[29] Valero C Lee M Hoen D Weiss K Kelly DW Adusumilli PS. Pretreatment neutrophil-to-lymphocyte ratio and mutational burden as biomarkers of tumor response to immune checkpoint inhibitors. Nat Commun (2021) 12(1):729. doi: 10.1038/s41467-021-20935-9 33526794 PMC7851155

[30] Dirican A Ekinci N Avci A Akyol M Alacacioglu A Kucukzeybek Y. The effects of hematological parameters and tumor-infiltrating lymphocytes on prognosis in patients with gastric cancer. Cancer Biomarkers. (2013) 13(1):11–20. doi: 10.3233/CBM-130331 23736017 PMC12928264

[31] Hirahara T Arigami T Yanagita S Matsushita D Uchikado Y Kita Y. Combined neutrophil-lymphocyte ratio and platelet-lymphocyte ratio predicts chemotherapy response and prognosis in patients with advanced gastric cancer. BMC Cancer (2019) 19(672):1–8. doi: 10.1186/s12885-019-5903-y PMC6615151 31286873

[32] Mao MJ Wei XL Sheng H Chi PD Liu YJ Huang XY. C-reactive protein/albumin and neutrophil/lymphocyte ratios and their combination predict overall survival in patients with gastric cancer. Oncol Letters. (2017) 14(6):7417–24. doi: 10.3892/ol.2017.7179 PMC5755031 29344182

[33] Sun R Champiat S Dercle L Aspeslagh S Castanon E Limkin EJ. Baseline lymphopenia should not be used as exclusion criteria in early clinical trials investigating immune checkpoint blockers (PD-1/PD-L1 inhibitors). Eur J Cancer. (2017) 84:202–11. doi: 10.1016/j.ejca.2017.07.033 28826073

[34] Gou M Qu T Wang Z Yan H Si Y Zhang Y. Neutrophil-to-Lymphocyte ratio (NLR) predicts PD-1 inhibitor survival in patients with metastatic gastric cancer. J Immunol Res (2021) 2021:2549295. doi: 10.1155/2021/2549295 34993252 PMC8727102

[35] Danielle Benedict S Lucero JA Dennis Lee S. Prognostic utility of baseline neutrophil-to-lymphocyte ratio in patients receiving immune checkpoint inhibitors: a review and meta-analysis. OncoTargets Ther (2018) 11:955–65. doi: 10.2147/OTT.S153290 PMC5827677 29503570

[36] Wang TT Zhao YL Peng LS Chen N Chen W Lv YP. Tumour-activated neutrophils in gastric cancer foster immune suppression and disease progression through GM-CSF-PD-L1 pathway. Gut (2017) 66(11):1900–11. doi: 10.1136/gutjnl-2016-313075 PMC5739867 28274999

[37] Hedrick CC Malanchi I. Neutrophils in cancer: heterogeneous and multifaceted. Nat Rev Immunol (2022) 22(3):173–87. doi: 10.1038/s41577-021-00571-6 34230649

[38] Deng QW He BS Liu X Yue J Ying HQ Pan YQ. Prognostic value of pre-operative inflammatory response biomarkers in gastric cancer patients and the construction of a predictive model. J Trans Med (2015) 13:66. doi: 10.1186/s12967-015-0409-0 PMC4343078 25885254

[39] Guthrie GJK Charles KA Roxburgh CSD Horgan PG McMillan DC Clarke SJ. The systemic inflammation-based neutrophil–lymphocyte ratio: Experience in patients with cancer. Crit Rev Oncology/Hematology. (2013) 88(1):218–30. doi: 10.1016/j.critrevonc.2013.03.010 23602134

[40] Moses K Brandau S. Human neutrophils: Their role in cancer and relation to myeloid-derived suppressor cells. Semin Immunol (2016) 28(2):187–96. doi: 10.1016/j.smim.2016.03.018 27067179

[41] Diaz-Montero CM Salem ML Nishimura MI Garrett-Mayer E Cole DJ Montero AJ. Increased circulating myeloid-derived suppressor cells correlate with clinical cancer stage, metastatic tumor burden, and doxorubicin–cyclophosphamide chemotherapy. Cancer Immunology Immunother (2009) 58(1):49–59. doi: 10.1007/s00262-008-0523-4 PMC3401888 18446337

[42] Zhang D He W Wu C Tan Y He Y Xu B. Scoring system for tumor-infiltrating lymphocytes and its prognostic value for gastric cancer. Front Immunol (2019) 10. doi: 10.3389/fimmu.2019.00071 PMC6361780 30761139

[43] Lee HE Chae SW Lee YJ Kim MA Lee HS Lee BL. Prognostic implications of type and density of tumour-infiltrating lymphocytes in gastric cancer. Br J Cancer. (2008) 99(10):1704–11. doi: 10.1038/sj.bjc.6604738 PMC2584941 18941457

[44] Li F Li C Cai X Xie Z Zhou L Cheng B. The association between CD8+ tumor-infiltrating lymphocytes and the clinical outcome of cancer immunotherapy: A systematic review and meta-analysis. EClinicalMedicine (2021) 41:101134. doi: 10.1016/j.eclinm.2021.101134 34585125 PMC8452798

[45] Tumeh PC Harview CL Yearley JH Shintaku IP Taylor EJM Robert L. PD-1 blockade induces responses by inhibiting adaptive immune resistance. Nature (2014) 515(7528):568–71. doi: 10.1038/nature13954 PMC4246418 25428505

[46] Pfirschke C Engblom C Rickelt S Cortez-Retamozo V Garris C Pucci F. Immunogenic chemotherapy sensitizes tumors to checkpoint blockade therapy. Immunity (2016) 44(2):343–54. doi: 10.1016/j.immuni.2015.11.024 PMC4758865 26872698

[47] Walsh SR Cook EJ Goulder F Justin TA Keeling NJ. Neutrophil-lymphocyte ratio as a prognostic factor in colorectal cancer. J Surg Oncol (2005) 91(3):181–4. doi: 10.1002/jso.20329 16118772

[48] Gomez D Morris-Stiff G Toogood GJ Lodge JP Prasad KR. Impact of systemic inflammation on outcome following resection for intrahepatic cholangiocarcinoma. J Surg Oncol (2008) 97(6):513–8. doi: 10.1002/jso.21001 18335453

[49] Cuzick J Dowsett M Pineda S Wale C Salter J Quinn E. Prognostic value of a combined estrogen receptor, progesterone receptor, ki-67, and human epidermal growth factor receptor 2 immunohistochemical score and comparison with the genomic health recurrence score in early breast cancer. J Clin Oncol (2011) 29(32):4273–8. doi: 10.1200/JCO.2010.31.2835 21990413

