# Supplementary material for: Association of inflammatory markers with survival in patients with advanced gastric cancer treated with immune checkpoint inhibitors combined with chemotherapy as first line treatment
Source: Front Oncol. 2022 Oct 28;12:1029960. doi: 10.3389/fonc.2022.1029960 (PMC9650180; doi:10.3389/fonc.2022.1029960)

Supplementary Material

# Supplementary Figures and Tables

## Supplementary Figures

**
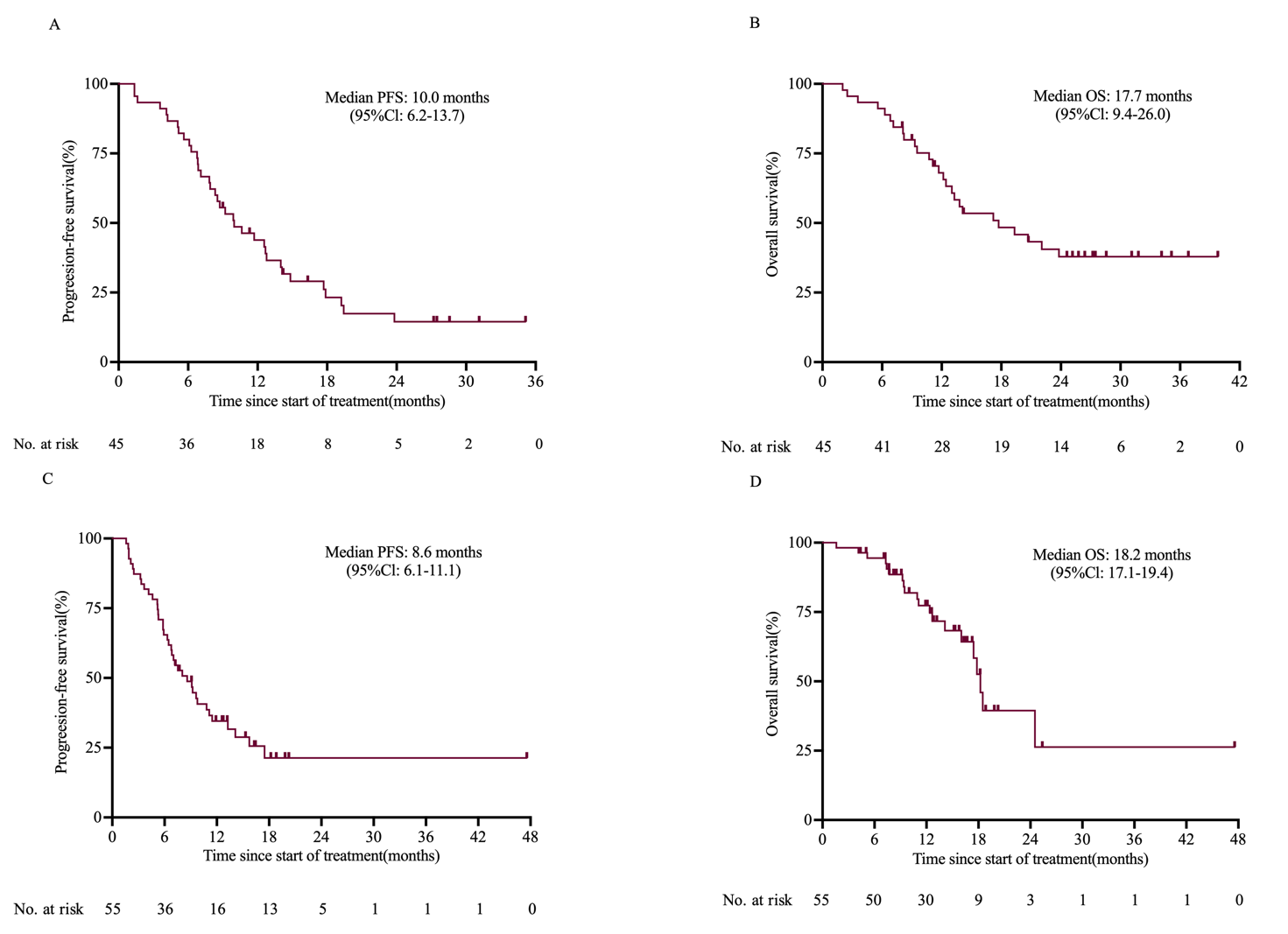
**

**Supplementary Figure 1.** Kaplan–Meier survival curves for (A) PFS, (B) OS of patients in the clinical cohort, (C) PFS and (D) OS of patients in the real-world cohort.

**
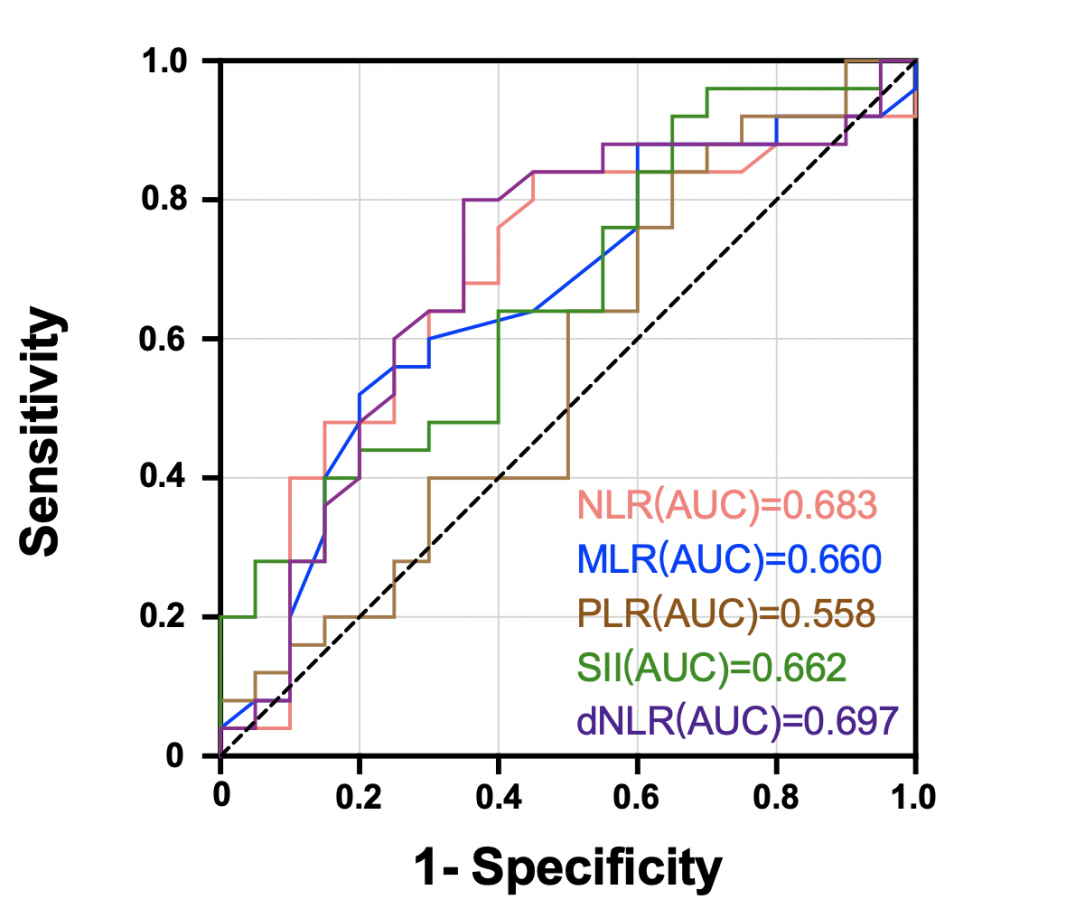
**

**Supplementary Figure 2.** Receiver operating characteristic curve for the inflammatory markers in the clinical cohort. *AUC* area under the receiver operating characteristics curve, *dNLR* derived neutrophil-to-lymphocyte ratio, *MLR* monocyte-to-lymphocyte ratio, *NLR* neutrophil-to-lymphocyte ratio, *PLR* platelet-to-lymphocyte ratio, *SII* systemic immune-inflammation index


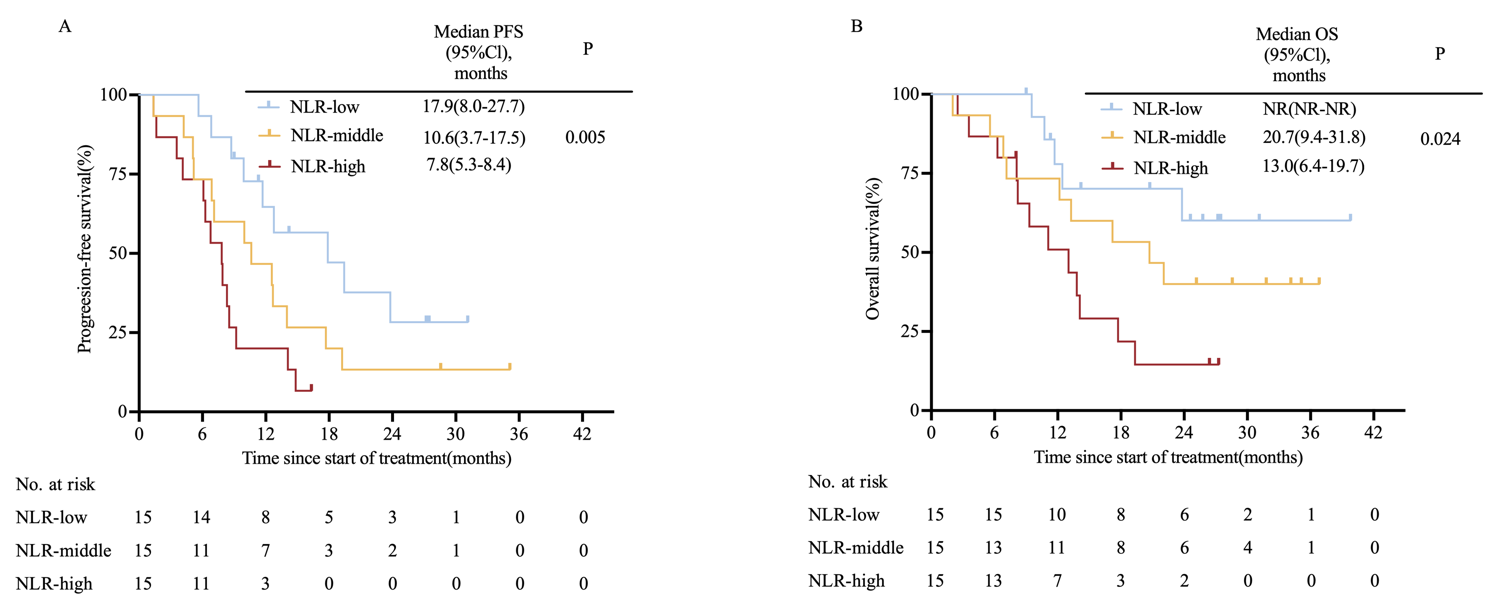


**Supplementary Figure 3.** Kaplan–Meier survival curves in the clinical trial cohort stratified by NLR for (A) PFS and (B) OS when divided the patients into three equal groups. *CI* confidence interval*, NLR* neutrophil-to-lymphocyte ratio*, OS* overall survival*, PFS* progression-free survival


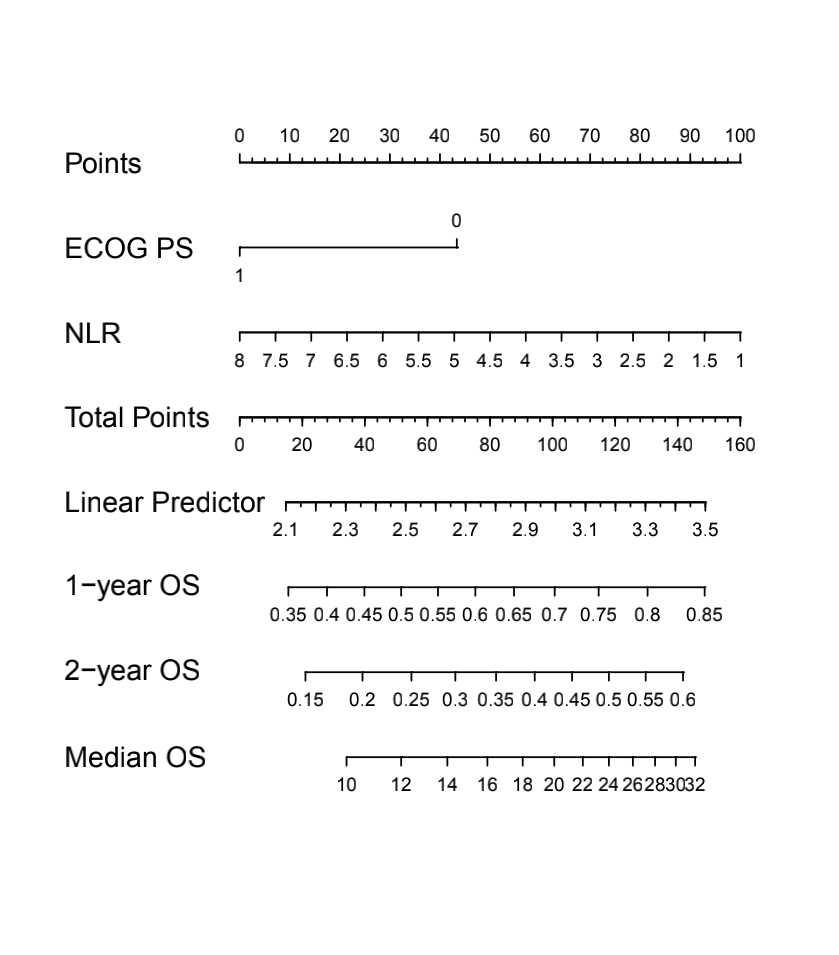


**Supplementary Figure 4.** Nomogram for predicting 1-year, 2-year, and median overall survival of advanced gastric cancer patients in the clinical trial cohort. *OS* overall survival, *NLR* neutrophil-to-lymphocyte ratio*, ECOG PS* Eastern Cooperative Oncology Group Performance Status.

**
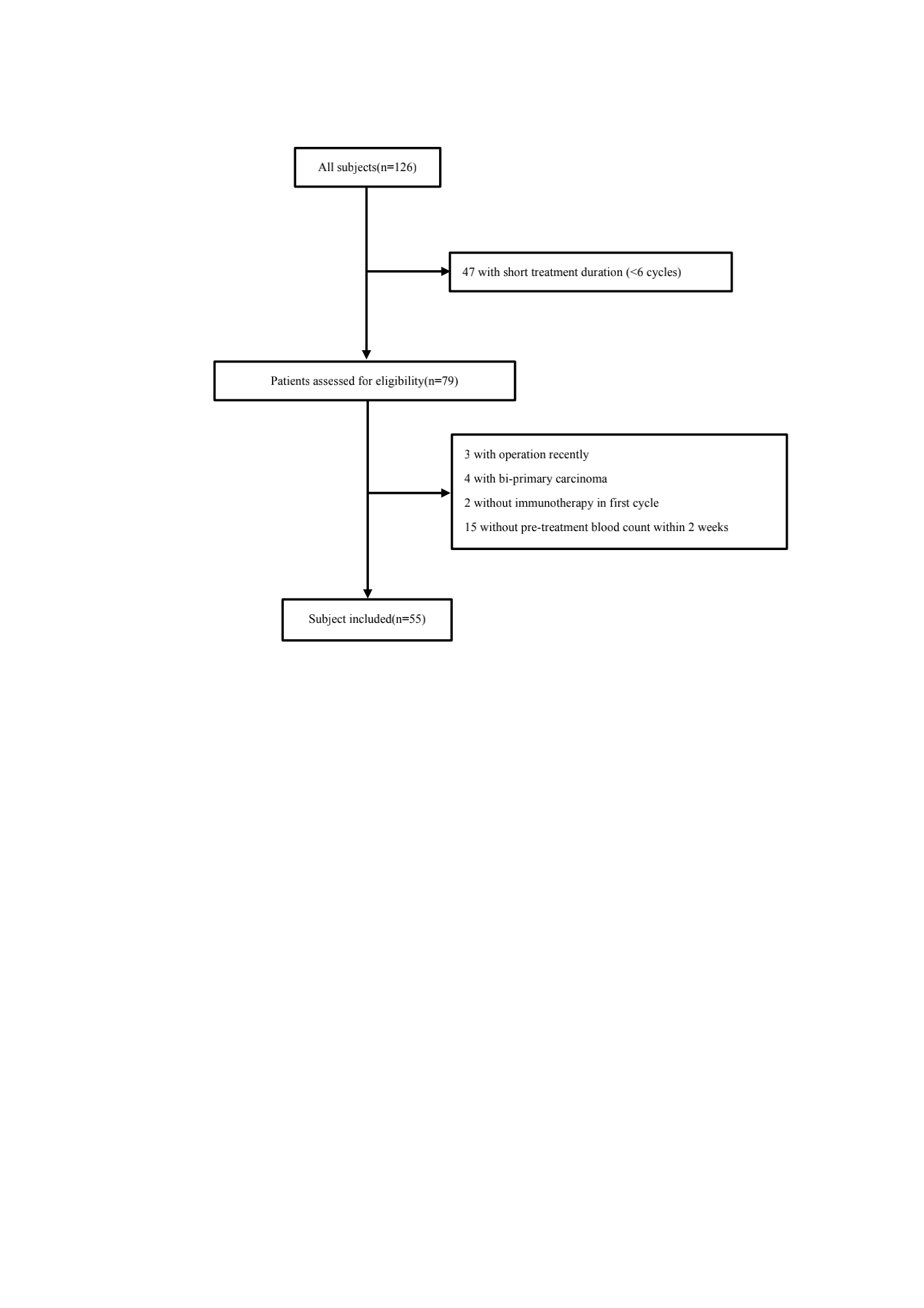
**

**Supplementary Figure 5.** Flowchart presenting the steps of inclusion and exclusion of subjects in the real-world cohort.

**
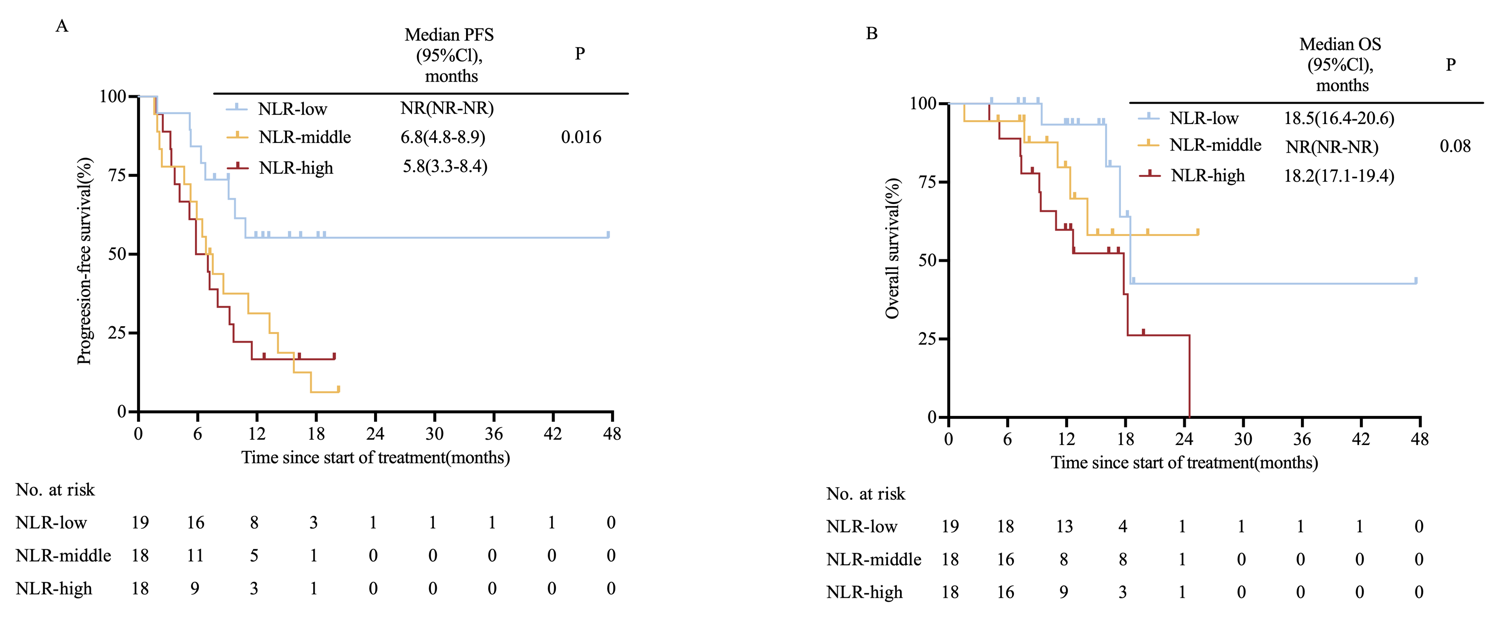
**

**Supplementary Figure 6.** Kaplan-Meier survival curves in the real-world cohort stratified by NLR for (A) PFS and (B) OS when divided the patients into three equal groups. *CI* confidence interval*, NLR* neutrophil-to-lymphocyte ratio*, OS* overall survival*, PFS* progression-free survival

**
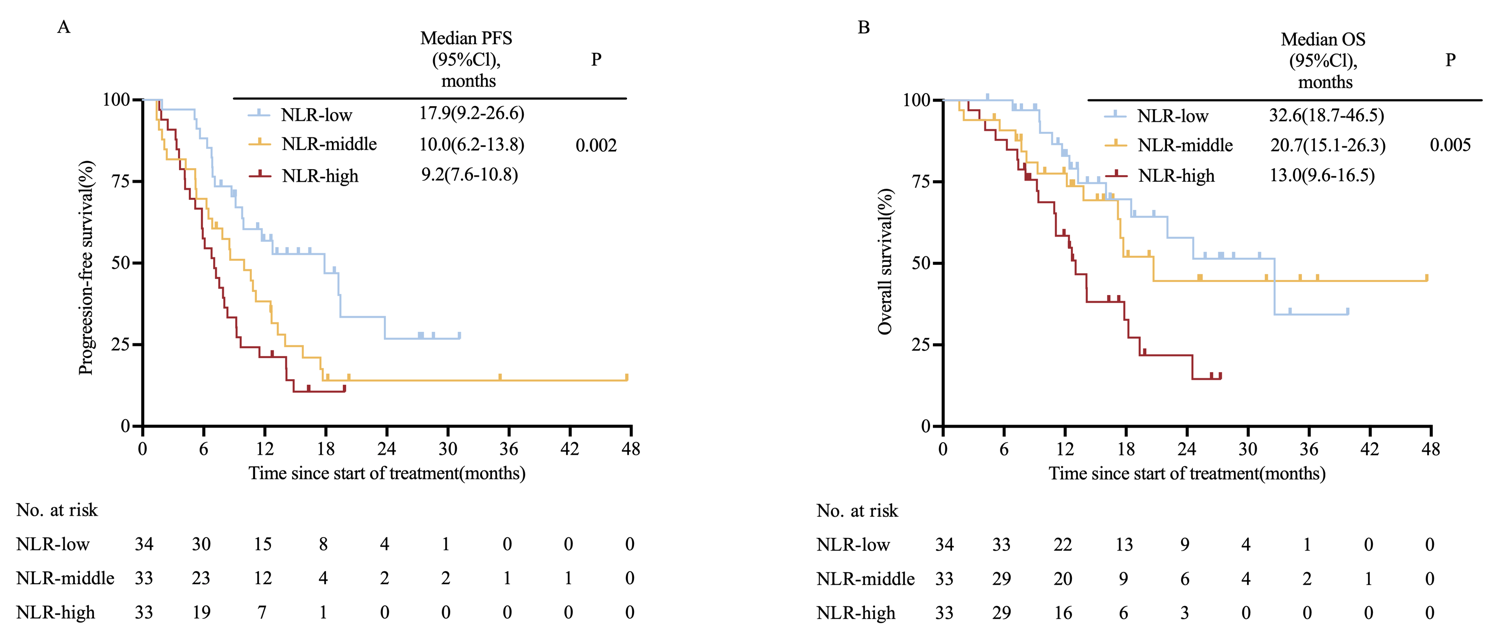
**

**Supplementary Figure 7.** Kaplan-Meier survival curves in the pooled cohort stratified by NLR for (A) PFS and (B) OS when divided the patients into three equal groups. *CI* confidence interval*, NLR* neutrophil-to-lymphocyte ratio*, OS* overall survival*, PFS* progression-free survival

## Supplementary Tables

**Supplementary Table 1.** Chemotherapy and immunotherapy regimens in the clinical trial cohort and real-world cohort.

| Characteristics | Clinical trial cohort(n=45) | Real-world cohort(n=55) | P-value |
| --- | --- | --- | --- |
| Chemotherapy |  |  | <0.001 |
| XELOX | 44(98%) | 9(16%) |  |
| SOX | 0(0%) | 17(31%) |  |
| FOLFOX | 1(2%) | 7(13%) |  |
| SPA | 0(0%) | 21(38%) |  |
| Others | 0(0%) | 1(2%) |  |
| Immunotherapy |  |  | <0.001 |
| Camrelizumab | 13(29%) | 17(31%) |  |
| Sintilimab | 17(38%) | 20(36%) |  |
| Tislelizumab | 0(0%) | 13(24%) |  |
| Toripalimab | 0(0%) | 1(2%) |  |
| CS1001 | 1(2%) | 0(0%) |  |
| Envafolimab | 1(2%) | 0(0%) |  |
| HX008 | 13(29%) | 0(0%) |  |

*FOLFOX* oxaliplatin plus fluorouracil and leucovorin, *SOX* S-1 plus oxaliplatin, *SPA* S-1 plus paclitaxel, *XELOX* oxaliplatin plus capecitabine

**Supplementary Table 2.** Efficiency in the clinical trial cohort and real-world cohort.

| Parameter | Clinical trial cohort(n=45) | Real-world cohort(n=55) | Pooled cohort(n=100) | |
| --- | --- | --- | --- | --- |
| Progression-free survival |  |  |  |  |
| Median(95%CI), months | 10.0(6.2-13.7) | 8.6(6.1-11.1) | 9.2(7.6-10.8) | |
| Overall survival |  |  |  | |
| Median(95%CI), months | 17.7(9.4-26.0) | 18.2(17.1-19.4) | 18.2(15.0-21.5) | |
| Best overall response, n (%) |  |  |  | |
| CR | 8(17.7) | 3(5.5) | 11(11.0) | |
| PR | 25(55.5) | 27(49.1) | 52(52.0) | |
| SD | 11(24.4) | 17(30.9) | 28(28.0) | |
| PD | 1(2.2) | 8(14.5) | 9(9.0) | |
| ORR | 33(73.3) | 30(54.5) | 57(57.0) | |
| DCR | 44(97.7) | 47(85.5) | 91(91.0) | |
| 1 year survival rate, % | 62.2 | 54.5 | 58.0 | |

*CR* complete response, *DCR* disease control rate, *ORR* objective response rate, *PD* progressive disease, *PR* partial response, *SD* stable disease

**Supplemental Table 3. Summary of treatment-related adverse events in the clinical trial cohort.**

|  | Grade 1-2 | Grade 3 | Grade 4 |
| --- | --- | --- | --- |
| Any TRAE | 45(100%) | 27(60%) | 4(9%) |
| Anemia | 27(60%) | 4(9%) | 0(0%) |
| Hepatic enzyme increased | 23(51%) | 0(0%) | 0(0%) |
| White blood cell count decreased | 20(44%) | 3(7%) | 0(0%) |
| Neutrophil count decreased | 20(44%) | 7(16%) | 1(2%) |
| Platelet count decreased | 15(33%) | 9(20%) | 3(7%) |
| Rash | 13(29%) | 0(0%) | 0(0%) |
| Peripheral neuropathy | 12(27%) | 2(4%) | 0(0%) |
| Vomiting | 12(27%) | 0(0%) | 0(0%) |
| Urine protein increased | 12(27%) | 0(0%) | 0(0%) |
| Thyroid dysfunction | 11(24%) | 0(0%) | 0(0%) |
| Nausea | 10(22%) | 0(0%) | 0(0%) |
| RCCEP | 9(20%) | 1(2%) | 0(0%) |
| Fatigue | 7(16%) | 2(4%) | 0(0%) |
| Decreased appetite | 7(16%) | 0(0%) | 0(0%) |
| Hypertension | 1(2%) | 6(13%) | 0(0%) |
| Hand-foot syndrome | 5(11%) | 1(2%) | 0(0%) |

Data are n (%). Any-grade events in 10% or more of treated patients. *RCCEP* reactive cutaneous capillary endothelial proliferation, *TRAE* treatment-related adverse event.

**Supplemental Table 4.** The subsequent treatment of patients in the real-world cohort.

| Regimens | Patients (n=30) |
| --- | --- |
| Platinum-based chemotherapy | 5(17%) |
| Paclitaxel-based chemotherapy | 11(37%) |
| Irinotecan | 7(23%) |
| Targeted therapy | 18(60%) |
| Apatinib | 11(37%) |
| Bevacizumab | 2(7%) |
| AK109 | 2(7%) |
| Trastuzumab | 1(3%) |
| Fruquintinib | 1(3%) |
| Others | 2(7%) |
| Immunotherapy | 19(63%) |
| Radiation therapy | 0(0%) |

**Supplementary Table 5.** Univariate and multivariate analyses of the association between baseline characteristics and survival in the real-world cohort

| Variable | PFS | | OS | |
| --- | --- | --- | --- | --- |
|  | Univariate analysis | Multivariate analysis | Univariate analysis | Multivariate analysis |
|  | HR (95% CI), P | HR (95% CI), P | HR (95% CI), P | HR (95% CI), P |
| Age (≥60 vs <60) | 0.58(0.31-1.11), P=0.098 | - | 0.67(0.27-1.67), P=0.391 | - |
| Gender (female vs male) | 0.72(0.33-1.59), P=0.415 | - | 1.24(0.48-3.20), P=0.655 | - |
| ECOG PS (1 or 2 vs 0) | 1.51(0.79-2.86), P=0.210 | P=0.568 | 1.09(0.45-2.63), P=0.852 | P=0.530 |
| Liver metastasis (yes vs no) | 0.93(0.49-1.74), P=0.811 | - | 0.84(0.34-2.07), P=0.696 | - |
| Peritoneum metastasis (yes vs no) | 0.81(0.38-1.70), P=0.574 | - | 0.84(0.28-2.53), P=0.753 | - |
| Differentiation (poor vs moderate-well) | 1.65(0.79-3.43), P=0.180 | P=0.559 | 2.30(0.76-6.92), P=0.139 | P=0.657 |
| History of operation (yes vs no) | 1.81(0.92-3.54), P=0.085 | - | 1.10(0.43-2.81), P=0.840 | - |
| History of smoke (yes vs no) | 1.05(0.55-2.00), P=0.884 | - | 0.56(0.21-1.47), P=0.240 | - |
| History of alcohol (yes vs no) | 1.31(0.67-2.55), P=0.434 | - | 0.70(0.25-1.95), P=0.489 | - |
| NLR (≥3.85 vs <3.85) | 2.21(1.16-4.21), P=0.016 | 2.67(1.35-5.27), P=0.005 | 2.94(1.12-7.72), P=0.028 | 3.69(1.40-9.11), P=0.008 |
| MLR (≥0.35 vs <0.35) | 2.10(1.04-4.24), P=0.039 | P=0.237 | 6.12(1.41-26.65), P=0.016 | P=0.152 |
| PLR (≥214.08 vs <214.08) | 2.07(1.08-3.96), P=0.028 | P=0.148 | 2.50(1.02-6.15), P=0.046 | P=0.232 |
| SII (≥1154.67 vs <1154.67) | 1.81(0.95-3.46), P=0.072 | - | 2.19(0.91-5.27), P=0.080 | - |
| dNLR (≥2.45 vs <2.45) | 1.74(0.91-3.31), P=0.095 | - | 1.51(0.60-3.79), P=0.383 | - |

*CI* confidence interval*, dNLR* derived neutrophil-to-lymphocyte ratio*, ECOG PS* Eastern Cooperative Oncology Group Performance Status*, HR* hazard ratio*, MLR* monocyte-to-lymphocyte ratio*, NLR* neutrophil-to-lymphocyte ratio*, OS* overall survival*, PLR* platelet-to-lymphocyte ratio, *PFS* progression-free survival*, SII* systemic immune-inflammation index

**Supplemental Table 6.** Efficacy in inflammatory marker-low group and inflammatory marker-high group in the pooled cohort.

| Parameter | Inflammatory marker-low | Inflammatory marker-high | P-value |
| --- | --- | --- | --- |
| NLR | n=59 | n=41 |  |
| Progression-free survival |  |  | <0.001 |
| Median(95%CI), months | 12.7(9.1-16.2) | 7.0(5.4-8.6) |  |
| Overall survival |  |  | 0.001 |
| Median(95%CI), months | 32.6(14.9-50.3) | 13.8(12.1-15.6) |  |
| Best overall response, n (%) |  |  | 0.395 |
| CR | 9(15.3) | 2(4.9) |  |
| PR | 28(47.5) | 24(58.5) |  |
| SD | 17(28.8) | 11(26.8) |  |
| PD | 5(8.5) | 4(9.8) |  |
| ORR | 37(62.7) | 24(63.4) | 0.943 |
| DCR | 54(91.5) | 51(90.2) | 1.000 |
| 1 year survival rate, % | 62.7 | 51.2 | 0.252 |
| MLR | n=43 | n=57 |  |
| Progression-free survival |  |  | 0.001 |
| Median(95%CI), months | 12.8(6.0-19.6) | 7.5(6.3-8.8) |  |
| Overall survival |  |  | 0.002 |
| Median(95%CI), months | 32.6(NR-NR) | 14.1(8.4-19.9) |  |
| Best overall response, n (%) |  |  | 0.084 |
| CR | 7(16.3) | 5(8.8) |  |
| PR | 17(39.5) | 35(61.4) |  |
| SD | 16(37.2) | 11(19.3) |  |
| PD | 3(7.0) | 6(10.5) |  |
| ORR | 24(55.8) | 40(70.2) | 0.139 |
| DCR | 40(93.0) | 51(89.5) | 0.794 |
| 1 year survival rate, % | 62.8 | 54.4 | 0.399 |
| PLR | n=65 | n=35 |  |
| Progression-free survival |  |  | 0.001 |
| Median(95%CI), months | 12.6(9.2-16.0) | 7.5(6.1-9.0) |  |
| Overall survival |  |  | 0.020 |
| Median(95%CI), months | 22.1(10.4-33.7) | 13.8(11.9-15.7) |  |
| Best overall response, n (%) |  |  | 0.892 |
| CR | 8(12.3) | 3(8.6) |  |
| PR | 34(52.3) | 18(51.4) |  |
| SD | 18(27.7) | 10(28.6) |  |
| PD | 5(7.7) | 4(11.4) |  |
| ORR | 42(64.6) | 21(60.0) | 0.648 |
| DCR | 60(92.3) | 31(88.6) | 0.798 |
| 1 year survival rate, % | 64.6 | 45.7 | 0.068 |
| SII | n=73 | n=27 |  |
| Progression-free survival |  |  | 0.004 |
| Median(95%CI), months | 10.8(7.8-13.9) | 7.2(5.9-8.5) |  |
| Overall survival |  |  | 0.079 |
| Median(95%CI), months | 20.7(14.3-27.1) | 14.1(8.6-19.6) |  |
| Best overall response, n (%) |  |  | 0.665 |
| CR | 9(12.3) | 2(7.4) |  |
| PR | 36(49.3) | 16(59.3) |  |
| SD | 20(27.4) | 8(29.6) |  |
| PD | 8(11.0) | 1(3.7) |  |
| ORR | 45(61.6) | 18(66.7) | 0.644 |
| DCR | 65(89.0) | 26(96.3) | 0.464 |
| 1 year survival rate, % | 58.9 | 55.6 | 0.763 |
| dNLR | n=55 | n=45 |  |
| Progression-free survival |  |  | 0.009 |
| Median(95%CI), months | 11.7(8.3-15.1) | 7.2(5.4-9.0) |  |
| Overall survival |  |  | 0.086 |
| Median(95%CI), months | 22.1(13.4-30.8) | 14.1(9.6-18.6) |  |
| Best overall response, n (%) |  |  | 0.204 |
| CR | 9(16.4) | 2(4.4) |  |
| PR | 25(45.5 | 27(60.0) |  |
| SD | 15(27.3) | 13(28.9) |  |
| PD | 6(10.9) | 3(6.7) |  |
| ORR | 34(61.8) | 29(64.4) | 0.787 |
| DCR | 49(89.1) | 42(93.3) | 0.699 |
| 1 year survival rate, % | 60.0 | 55.6 | 0.654 |

*dNLR* derived neutrophil-to-lymphocyte ratio*, MLR* monocyte-to-lymphocyte ratio*, NLR* neutrophil-to-lymphocyte ratio*, PLR* platelet-to-lymphocyte ratio, *SII* systemic immune-inflammation index

**Supplementary Table 7.** Univariate and multivariate analyses of the association between baseline characteristics and survival in the pooled cohort.

| Variable | PFS | | OS | |
| --- | --- | --- | --- | --- |
|  | Univariate analysis | Multivariate analysis | Univariate analysis | Multivariate analysis |
|  | HR (95% CI), P | HR (95% CI), P | HR (95% CI), P | HR (95% CI), P |
| Age (≥60 vs <60) | 0.53(0.33-0.85), P=0.008 | P=0.112 | 0.62(0.35-1.11), P=0.110 | - |
| Gender (female vs male) | 1.02(0.59-1.75), P=0.958 | - | 1.45(0.76-2.76), P=0.256 | - |
| ECOG PS (1 or 2 vs 0) | 2.02(1.33-3.07), P=0.001 | P=0.075 | 1.34(0.75-2.41), P=0.328 | P=0.564 |
| Liver metastasis (yes vs no) | 0.91(0.58-1.43), P=0.684 | - | 0.80(0.45-1.43), P=0.446 | - |
| Peritoneum metastasis (yes vs no) | 0.94(0.52-1.68), P=0.828 | - | 0.98(0.46-2.11), P=0.960 | - |
| Differentiation (poor vs moderate-well) | 1.64(1.01-2.69), P=0.048 | P=0.252 | 1.67(0.92-3.03), P=0.092 | P=0.491 |
| History of operation (yes vs no) | 1.77(1.04-2.99), P=0.034 | P=0.053 | 1.43(0.74-2.77), P=0.290 | - |
| History of smoke (yes vs no) | 1.24(0.76-2.02), P=0.388 | - | 0.86(0.44-1.67), P=0.653 | - |
| History of alcohol (yes vs no) | 1.29(0.77-2.14), P=0.322 | - | 0.99(0.50-1.96), P=0.978 | - |
| NLR (≥3.85 vs <3.85) | 2.42(1.51-3.86), P<0.001 | 2.75(1.61-4.71), P<0.001 | 2.64(1.46-4.80), P=0.001 | 3.25(1.76-6.00), P<0.001 |
| MLR (≥0.35 vs <0.35) | 2.28(1.40-3.72), P=0.001 | P=0.140 | 2.70(1.41-5.18), P=0.003 | P=0.382 |
| PLR (≥214.08 vs <214.08) | 2.18(1.35-3.52), P=0.001 | P=0.139 | 2.00(1.10-3.62), P=0.022 | P=0.384 |
| SII (≥1154.67 vs <1154.67) | 2.02(1.24-3.31), P=0.005 | P=0.533 | 1.72(0.93-3.16), P=0.083 | - |
| dNLR (≥2.45 vs <2.45) | 1.81(1.15-2.86), P=0.011 | P=0.275 | 1.65(0.93-2.94), P=0.090 | - |

*CI* confidence interval*, dNLR* derived neutrophil-to-lymphocyte ratio*, ECOG PS* Eastern Cooperative Oncology Group Performance Status*, HR* hazard ratio*, MLR* monocyte-to-lymphocyte ratio*, NLR* neutrophil-to-lymphocyte ratio*, OS* overall survival*, PLR* platelet-to-lymphocyte ratio, *PFS* progression-free survival*, SII* systemic immune-inflammation index


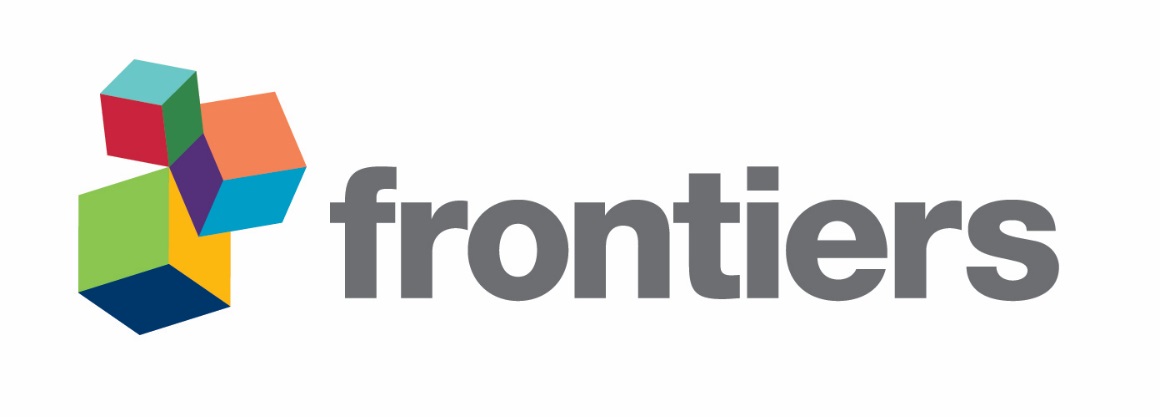

Supplement: Supplementary file 1 [file DataSheet_1.docx]
